# Investigation on the Interactions of NiCR and NiCR-2H with DNA

**DOI:** 10.1155/2010/619436

**Published:** 2010-06-30

**Authors:** Priyanka Chitranshi, Chang-Nan Chen, Patrick R. Jones, Jesika S. Faridi, Liang Xue

**Affiliations:** ^1^Department of Chemistry, University of the Pacific, Stockton, CA 95211, USA; ^2^Department of Applied Chemistry, Chaoyang University of Technology, Taichung 41349, Taiwan; ^3^T. J. L. School of Pharmacy and Health Sciences, University of the Pacific, Stockton, CA 95211, USA

## Abstract

We report here a biophysical and biochemical approach to determine the differences in interactions of NiCR and NiCR-2H with DNA. Our goal is to determine whether such interactions are responsible for the recently observed differences in their cytotoxicity toward MCF-7 cancer cells. Viscosity measurement and fluorescence displacement titration indicated that both NiCR and NiCR-2H bind weakly to duplex DNA in the grooves. The coordination of NiCR-2H with the N-7 of 2′-deoxyguanosine 5′-monophosphate (5′-dGMP) is stronger than that of NiCR as determined by ^1^H NMR. NiCR-2H, like NiCR, can selectively oxidize guanines present in distinctive DNA structures (e.g., bulges), and notably, NiCR-2H oxidizes guanines more efficiently than NiCR. In addition, UV and ^1^H NMR studies revealed that NiCR is oxidized into NiCR-2H in the presence of KHSO_5_ at low molar ratios with respect to NiCR (≤4).

## 1. Introduction

**Figure 1:**
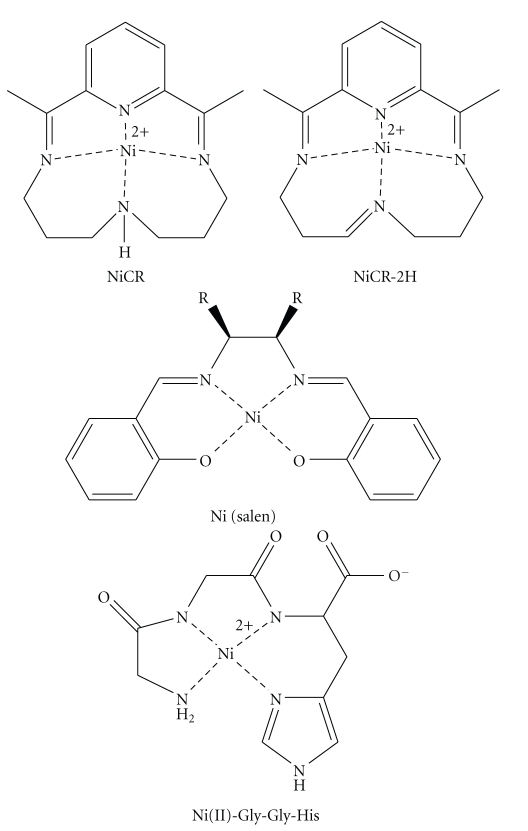
Representative nickel complexes.

Natural and synthetic nickel [especially Ni (II)] complexes (Figure 1) can oxidatively damage nucleic acids via redox reactions, resulting in direct strand breaks and modified bases (lesions) [1–5]. If not repaired properly, DNA lesions can be mutagenic and have been implicated in aging and diseases such as cancer [6, 7]. Therefore, nickel-containing complexes that oxidize DNA are of biological importance. A classic example is Ni(II)∙Gly∙Gly∙His, a naturally occurring metallopeptide, found in the N-terminal Cu (II) or Ni (II) chelating domain of the serum albumins [8], human sperm protamine P2a [9], and the histatins [3]. Its mechanism of action involves redox reactions of Ni (II) in the presence of exogenous chemical oxidants to produce a ligand- or metallopeptide-based radical, which subsequently abstracts hydrogen(s) from proximate DNA backbones to induce strand breaks [10, 11]. Over the years, synthetic nickel (II) complexes mimicking their natural counterparts have been developed and investigated for their oxidation of DNA. Bailly and coworkers and others showed that Ni(salen) coordinated complexes can form adducts with guanines in RNA or DNA via a phenolic radical [12, 13]. Burrows and coworkers studied NiCR that was formed by coordination of Ni (II) with 2,12-dimethyl-3,7,11,17-tetraazabicyclo-[11.3.1]-heptadeca-1,2,11,13,15-pentaene (CR) as a ligand [2, 14]. NiCR preferentially oxidizes guanine(s) in single-stranded nucleic acids, at the end of DNA duplexes, and in the DNA duplex regions where guanine residues do not adopt standard Watson-Crick base pairing. Exogenous oxidants such as oxone are required for such oxidation, and the oxidation is believed to involve an unstable Ni (III) complex intermediate [15, 16]. In addition, oxidation of DNA by NiCR cannot directly produce DNA strand breaks unless DNA is further treated with hot alkaline conditions (e.g., piperidine). The same investigators have also successfully utilized NiCR as a molecular probe for detecting unique DNA structures containing guanine(s) such as bulges, loops, and hairpins [14, 17, 18]. 

Although NiCR and other Ni (II) complexes as DNA damaging agents have been rigorously characterized, new biochemical properties keep emerging. A recent study of NiCR and its close structural analogue NiCR-2H revealed that NiCR-2H was cytotoxic (IC_50_: ~70–80 *μ*M) toward MCF-7 cancer cells in the absence of any exogenous oxidant while NiCR had no effect on cell growth [19]. In the same study, strand breaks in calf thymus (CT) and plasmid DNA by millimolar concentrations of NiCR were observed in the absence of any exogenous oxidant. The explanation for the observed differences in cytotoxicity remains unclear. In this paper, we focus on analyzing the binding modes of NiCR and NiCR-2H with duplex DNA, their coordination with the N7 of 5′-dGMP, the oxidation of NiCR by oxone, and the DNA cleavage efficacy of NiCR and NiCR-2H. Our goal is to determine if the differences in molecular interactions of NiCR and NiCR-2H with DNA are responsible for the observed differences in cytotoxicity in cultured cells.

## 2. Experimental

### 2.1. Materials and General Methods

Oligonucleotides were purchased from Fisheroligos (Pittsburgh, PA). NiCR and NiCR-2H were synthesized based on the previously published procedures [20, 21]. Unless otherwise specified, chemicals for synthesis were purchased from Aldrich (St. Louis, MO) or Fisher Scientific (Pittsburgh, PA) and used without further purification. Calf Thymus DNA (Code No.: MB-102-0100, Lot No.: 20471) was purchased from Rockland (Gilbertsville, PA). Viscosity data were collected using an Ostwald-type viscometer. ^1^H NMR spectra were collected on a JEOL ECA 600 MHz FT-NMR spectrometer (Redding, CA). UV spectra were collected on a Varian Cary 100 Bio UV-Vis spectrophotometer (Walnut Creek, CA). Fluorescence spectra were collected on a Perkin-Elmer LS 55 fluorescence spectrophotometer (Waltham, MA). Circular dichroism spectra were recorded on a JASCO J-810 spectropolarimeter (Easton, MD) using a quartz cuvette with a 1 cm optical path length. T4 polynucleotide kinase was obtained from New England Biolabs (Ipswich, MA). [*γ*-^32^P]-ATP was purchased from MP Biochemicals (Solon, OH). Quantification of 5′  ^32^P-labeled oligonucleotides was carried out using a Storm 860 phosphorimager and ImageQuant 5.1 software (Molecular Dynamics, Sunnyvale, CA). Cell medium and supplements (fetal bovine serum, L-glutamine, penicillin, streptomycin, amphotericin B) were acquired from Invitrogen (Carlsbad, CA). Cell Titer 96 AQueous One Solution (MTS) was purchased from Promega (San Luis Obispo, CA). Analysis of cell viability (the MTS assay) was carried out on a Thermo Scientific Multiskan Ex plate reader (Waltham, MA) and the number of cells (dye exclusion assay) was counted using a hemocytometer under a microscope. DNA labeling was performed by incubating [*γ*-^32^P]-ATP (30 *μ*Ci) and T4 polynucleotide kinase (20 units) in the presence of an oligonucleotide (10 pmol) at 37°C for 30 min. Unreacted [*γ*-^32^P]-ATP was removed using a MicroSpin G-25 column (IBI Scientific, Peosta, IA). Cells were maintained in advanced DMEM/F12 medium supplemented with 5% fetal bovine serum, L-glutamine and penicillin (50 IU/mL), streptomycin (50 *μ*g/mL) and amphotericin B at 37°C in a humid atmosphere containing 5%  CO_2_ and air.

### 2.2. Viscosity Experiments

Calf thymus (CT) DNA was dissolved in a mixture of sodium phosphate buffer (10 mM, pH 7.0) and NaCl (100 mM). The concentration of the DNA was determined by UV spectroscopy, using a molar extinction coefficient at 260 nm (12,800 M^−1^ cm^−1^ bp^−1^). Small aliquots of a concentrated stock solution of ethidium bromide (EB), NiCR, or NiCR-2H were added into a 2 mL of DNA solution (1 mM) in an Ostwald-type viscometer that was immersed in a thermostated water bath at 25°C to obtain the desired ligand/DNA ratios. After each addition, the solution was mixed by bubbling with N_2_. The time for the level of the liquid to pass between two marks on the viscometer was recorded using a stopwatch. The relative viscosities were calculated based on the published equations [22].

### 2.3. Oxidation of NiCR by *K* *H* *S* *O*_5_

KHSO_5_ (3 equivalent, 0.058 mmol or 10 equiv., 0.193 mmol) was added to an aqueous solution (400 *μ*L) of NiCR (10 mg) and incubated for 5 min. The reaction mixture was then evaporated to dryness and the residue was suspended in acetonitrile (500 *μ*L). After filtration, the acetonitrile solution was concentrated under vacuum. The residue was dissolved in CF_3_COOD and subjected to ^1^H NMR measurements. The spectra of NiCR and NiCR-2H in CF_3_COOD were also recorded.

### 2.4. Fluorescence Displacement Titration

CT DNA (5 *μ*M) and ethidium bromide (5 *μ*M) were premixed in a 2 mL of Tris-HCl buffer (10 mM) and NaCl (100 mM) and allowed to stand for 30 min at 25°C. Small aliquots of a stock solution of the ligand (NiCR, NiCR-2H, or 9-aminoacridine) were added into the DNA-EB complex solution until the 1 : 1 ligand/DNA ratio was reached. After each addition, the mixture was incubated for 15 min at 25°C prior to the fluorescence analysis (Ex: 546 nm and Em: 605 nm). The fluorescence spectra of the ligands in the absence of CT DNA and EB were measured and used as blanks.

### 2.5. ^1^ *H* NMR Analysis of NiCR with 5′-dGMP

The 5′-dGMP stock solution (100 mM) was prepared by dissolving 5′-dGMP in D_2_O followed by lyophilization to dryness twice and then redissolving in D_2_O. A NiCR-2H stock solution (36.37 mM) was also prepared in D_2_O. Each individual sample (600 *μ*L) was prepared by mixing the 5′-dGMP stock solution with the NiCR-2H stock to make a final concentration of 50 mM 5′-dGMP and a desired ligand/5′-dGMP ratio ranging from 0 to 0.33. The samples were then subjected to ^1^H NMR measurements.

### 2.6. UV Analysis of Oxidation of NiCR

The oxone solution was freshly prepared in water prior to the reactions. Based on the molecular formula of oxone (2KHSO_5_∙KHSO_4_∙K_2_SO_4_), the concentration of KHSO_5_ was two fold higher than that of oxone. NiCR (1 mM) in water was mixed with a KHSO_5_ stock to obtain a desired ligand/KHSO_5_ ratio ranging from 0 to 10. The absorbance of each individual mixture was recorded from 350–800 nm.

### 2.7. Analysis of Reactions of NiCR and NiCR-2H with DNA Containing a Bulge by Denaturing PAGE

All experiments were carried out in triplicate. A 15-mer oligodeoxynucleotide duplex (**1**, 10 *μ*M) mixed with a small amount of 5′  ^32^P-labeled **1** in phosphate buffer (10 mM, pH 7.0) and NaCl (100 mM) was prepared by heating at 90°C for 5 min and then slowly cooling down to 25°C, and incubated at 4°C overnight. 5′  ^32^P-labeled **1** was added such that the radiation of DNA solution was approximately 20,000 cpm/*μ*L. The DNA (1 *μ*M) was incubated in a mixture (10 *μ*L) of phosphate buffer (10 mM, pH 7.0), NaCl (100 mM), NiCR or NiCR-2H (30 *μ*M), and KHSO_5_ (ranging from 0–1 mM) at 25°C for 30 min. After quenching the reaction by addition of NaHSO_3_ (1 *μ*L of 200 mM stock), the DNA products were precipitated from NaOAc (0.3 M) and EtOH at −80°C. After centrifugation, removal of the supernatant, and drying, the residue was treated with 10 *μ*L of piperidine (1 M) at 90°C for 20 min, concentrated, and resuspended in formamide loading buffer (5 *μ*L). Analytical oligonucleotide separations were carried out using 20% polyacrylamide denaturing gel (5% crosslink, 45% urea (w/w)).

### 2.8. The MTS Assay

All the experiments were carried out in triplicate. The nickel complex (NiCR or NiCR-2H) was predissolved in the medium and filtered using 0.2 micron sterile filter. The medium solution (50 *μ*L) containing ~3 × 10^3^ cells was added into a 96-well microtiter plate and incubated in 5% CO_2_ incubator for 24 h. This would allow cells to reach ~90% confluence. The nickel complex solution (50 *μ*L) was then added into the microtiter plate to reach a desired concentration and incubated for 72 h at 37°C. After incubation, a Cell Titer 96 AQueous One Solution (20 *μ*L) was added to each individual well. Quantification of viable cells was done by measuring the absorbance at 492 nm using a plate reader. Untreated cells and media with no cells were used as controls.

To calculate the viability (%), the following equation was used.

(1) $$\text{Viability}\;\;\left(\text{\%}\right)=\frac{\left(A_{\text{treated}}\right)-\left(A_{\text{media}}\right)}{\left(A_{\text{untreated}}\right)-\left(A_{\text{media}}\right)}\times 100\text{\%}.$$

*A*_treated_: The absorbance of the solution containing treated cells, *A*_media_: The absorbance of the media, *A*_untreated_: The absorbance of the solution containing untreated cells.

### 2.9. Statistical Analysis

Minitab 15.0 software was used to determine the statistical significance. Two-sample Student's *t* *-*test was performed to show statistically significant (*P* < .05) and insignificant (*P* > .05) data.

## 3. Results and Discussion

### 3.1. NiCR-2H Is More Cytotoxic to MCF-7 Cells than NiCR

**Figure 2:**
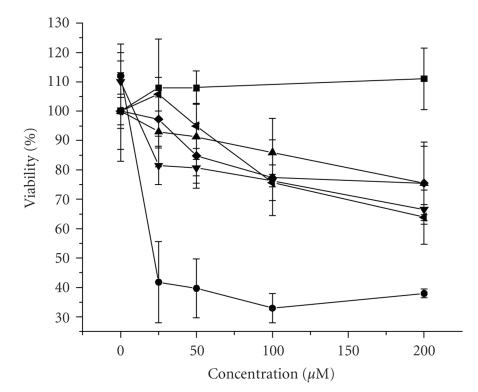
Effect of NiCR and NiCR-2H on the viability of cancer cells. NiCR with MCF-7 (▪), NiCR with HeLa (♦), NiCR with A-549 (▲), NiCR-2H with MCF-7 (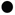), NiCR-2H with HeLa (◂), and NiCR-2H with A-549 (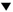).

Our initial attempts were to find a general trend of cytotoxicity of NiCR-2H toward different cancer cells. Three cancer cell lines, HeLa (human cervical cancer), A549 (human lung cancer), and MCF-7 (human breast adenocarcinoma) were chosen for study. Dye exclusion staining and the MTS assay were used to determine the cytotoxicity. In a dye exclusion test, dead cells are blue because they cannot exclude the dye molecule (trypan blue) in the media. In a MTS assay, the absorbance of a reduction product (formazan) from a tetrazolium salt (MTS) is determined spectroscopically. Only live cells are able to release active reductases that catalyze the reduction reaction; therefore, the absorbance of formazan is proportional to the number of live cells in culture. The results from the MTS assay are shown in Figure 2. NiCR-2H is more cytotoxic (IC_50_: 20 *μ*M) toward MCF-7 cells than NiCR in a statistically significant manner, and NiCR barely has any effect on inhibition of the growth of MCF-7 cells. These observations are consistent with the previous report [19]. It is noteworthy that our observed IC_50_ value for NiCR-2H is less than previously reported. The IC_50_ values of NiCR and NiCR-2H for HeLa and A549 cells could not be determined within the concentration range used for the two nickel complexes (Figure 2). Both NiCR and NiCR-2H at high concentrations became slightly cytotoxic to HeLa and A549 cells. The reductions in cell viability for both HeLa and A549 with NiCR (200 *μ*M) and NiCR-2H (200 *μ*M) were 25% and 35%, respectively. Surprisingly, dye exclusion staining resulted in 90–100% cell viability for all cells at all NiCR and NiCR-2H concentrations(See Table S-1 of the Supplementary Material available online on doi: 10.115512010/619436). The disagreement between cytotoxicity of known drugs and dye exclusion results has been previously reported in [23–25]. Dye exclusion has been used as an indicator of cell membrane integrity [26]. Dead cells (e.g., the reproductively dead) that do not have major membrane damage are known to exclude the dye molecule such as trypan blue. On the other hand, the MTS assay relies on active reductases released by live cells in the media and is probably more suitable for our cytotoxicity studies. However, dye exclusion staining was useful to explain the absorbance readings representing over 100% cell viability in the MTS assay for NiCR with MCF-7 cells (Figure 2). The overmeasured absorbance must result from the cell proliferation by NiCR as determined by dye exclusion staining. Nevertheless, the results from the MTS assay have undoubtedly confirmed that NiCR-2H is more cytotoxic to MCF-7 cells than NiCR and has little effect on HeLa and A549. In order to understand the differences in cytotoxicity, we in the present paper have compared the differences in molecular interactions of NiCR and NiCR-2H with duplex DNA. The results obtained using biophysical and biochemical methods are described below. 

### 3.2. Both NiCR and NiCR-2H Bind Weakly in the DNA Grooves

The pioneering work by Burrows and coworkers revealed a minimum binding of NiCR to duplex DNA [18]. Later, studies by Hellmann-Blumberg's laboratory suggested the binding of NiCR and NiCR-2H to duplex DNA is either intercalation or groove binding, which was not clearly distinguished [19]. Both NiCR and NiCR-2H were found to significantly displace ethidium bromide out of duplex DNA [19], suggesting relatively strong binding of NiCR and NiCR-2H to duplex DNA. 

**Figure 3:**
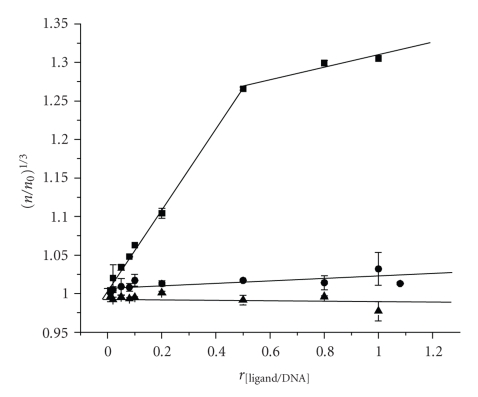
Viscosity changes of calf thymus DNA (1 mM) with increasing concentration (0.1 mM to 1 mM) of ethidium bromide (▪), NiCR-2H (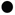), and NiCR (▴), respectively. Experimental conditions: 10 mM phosphate buffer (pH 7.0) at 25°C.

Because molecular interactions of NiCR and NiCR-2H with duplex DNA had not been fully investigated until the present work, we have been able to characterize these interactions using simple and reliable procedures of viscosity measurement [27, 28]. Noncovalent binding of small molecules to duplex DNA occurs mainly via either intercalation or groove binding mode [29]. The viscosity of a duplex DNA solution varies proportionally with the concentration of an intercalator due to the elongation of DNA length by intercalation. Groove binders have no effect on DNA length; therefore, the viscosity of a DNA solution is unaffected by the groove binding. In our experiments, a calf thymus DNA solution (1 mM in base pairs) was titrated with the molecule of interest varied over the range of 0–1.1 mM. The viscosity of the CT DNA solution in the presence of NiCR or NiCR-2H remained unchanged even when the ligand/DNA ratio was raised up to 1.1 (Figure 3). In contrast, the viscosity of the DNA solution varied linearly with the concentration of ethidium bromide (a known intercalator) until the EB/DNA ratio was above 0.5, at which a clear plateau was observed (Figure 3). The formation of the plateau indicates that all the possible binding sites in DNA are saturated by EB at this EB/DNA ratio, which can be explained with the neighbor exclusion principle [30]. The use of EB here was to provide a benchmark for this study. Our experiments lead us to conclude that DNA intercalation is not the major mode on non-covalent interaction of NiCR or NiCR-2H with duplex DNA. 

**Figure 4:**
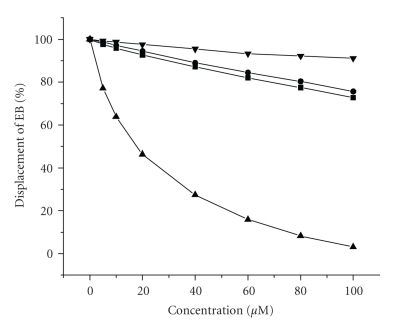
Fluorescence displacement titration of a preformed EB-DNA solution (5 *μ*M) with 9-aminoacridine (▴), NiCR (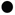), NiCR-2H (▪), and water (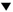), respectively. Experimental conditions: 10 mM phosphate buffer (pH 7.0) at 25°C.

The interactions between small molecules and DNA can also be determined spectroscopically [1, 31]. When a preformed EB-DNA complex in solution (5 *μ*M) was titrated with 9-aminoacridine (a known competitive DNA intercalator), a decrease in fluorescence (Ex: 546 nm and Em: 605 nm) of the solution was clearly observed. A dose-dependent reduction in fluorescence was found with up to 97% reduction at 100 *μ*M 9-aminoacridine as compared to the control (Figure 4). In contrast to 9-aminoacridine, titrating NiCR or NiCR-2H into a preformed EB-DNA complex solution (5 *μ*M) only gave rise to a subtle decrease in fluorescence (Figure 4). An approximate 16% fluorescence reduction was observed in the presence of NiCR (100 *μ*M) or NiCR-2H (100 *μ*M) after subtracting the background intensity (Figure 4), suggesting that both nickel complexes weakly displace ethidium bromide out of DNA. The rank order for binding given by the C_50_ values (drug concentrations required to affect a 50% reduction of the initial bound EB fluorescence) is 9-aminoacridine (~19 *μ*M) > NiCR-2H (~278 *μ*M) > NiCR (~327 *μ*M). The C_50_ values for NiCR-2H and NiCR were obtained by extending the titration curves to reach the theoretical 50% reduction. A quantitative analysis [32] of these C_50_ values in conjunction with the previously published binding constant of EB (10^7^ M^−1^) and the EB concentration (5 *μ*M) gives the apparent binding constant of 2.6 × 10^6^ M^−1^, 1.8 × 10^5^ M^−1^, and 1.5 × 10^5^ M^−1^ for 9-aminoacridine, NiCR-2H, and NiCR, respectively. The binding constant of 9-aminoacridine to CT DNA derived from the fluorescence titration experiments (Figure 4) is compatible with a previously reported value [33]. However, the binding constants of NiCR-2H and NiCR could be overestimated because the binding site sizes of NiCR-2H and NiCR should not be the same as EB (groove binding versus intercalation) [34]. Our results revealed that the ability to displace EB out of duplex DNA by NiCR or NiCR-2H is much weaker than previously reported in [19], and our data are actually in line with Burrows' conclusion. The disagreement between our fluorescence titration results and Hellmann-Blumberg's is probably due to the use of different salt concentrations in the experiments. The salt concentration used in our experiments was 100 mM, which is 10-fold more than that used by the other group and is commonly used for *in vitro* studies. It is known that cations (e.g., Na^+^, K^+^) can prevent positively charged species (NiCR and NiCR-2H in our case) from binding to DNA due to the electrostatic repulsion. Hence, the binding of NiCR or NiCR-2H with DNA at 100 mM NaCl is expected to be weaker than that in 10 mM NaCl. Based on the results of viscosity and fluorescence titration, we conclude that NiCR and NiCR-2H bind weakly to duplex DNA in the grooves under physiological conditions. 

Further evidence for the weak binding comes from the UV denaturation experiments. The melting temperatures of a 22-mer (AT tracts) or a 16-mer (mixed base) DNA oligonucleotide duplex are independent of the concentration of NiCR or NiCR-2H (See Figure S-1–Figure S-3 of the Supplementary Material), suggesting that both complexes cannot stabilize duplex DNA probably due to the minimal binding. Collectively, the little quantitative differences in the binding of NiCR and NiCR-2H with DNA lead us to conclude that the binding of the two with DNA should not be responsible for the differences in cytotoxicity. 

### 3.3. NiCR-2H Coordinates More Strongly to the N-7 in 5′-dGMP than NiCR

**Scheme 1:**
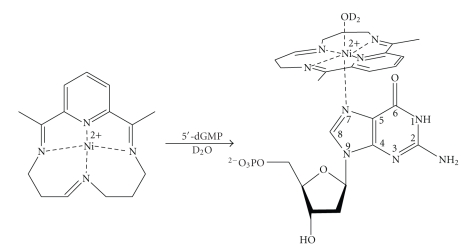
Coordination of NiCR-2H with 5′-dGMP.

**Figure 5:**
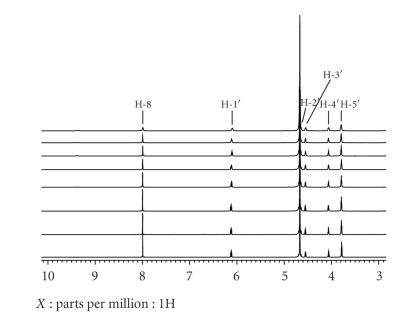
^1^H NMR spectra of 5′-dGMP (50 mM) in the presence of NiCR-2H at different concentrations (from bottom to top: 0, 0.5, 1, 2.5, 4, 7.5, 10, and 15 mM, resp.).

Metal complexes are known to coordinate with guanine because the N-7 position of guanine is the most nucleophilic site [35]. The coordination of NiCR or NiCR-2H with ligands (such as H_2_O and guanine) changes its geometry from square planar to octahedral (Scheme 1). The coordinated complexes become paramagnetic, perturbing the chemical shift and the relaxation of the proximate protons of guanine. In our experiments, 5′-dGMP (Scheme 1) was used as a model compound to coordinate with NiCR-2H. Each solution in D_2_O containing 5′-dGMP (50 mM) and NiCR-2H varied over the range of 0–16.5 mM was individually prepared to guarantee an accurate 5′-dGMP/NiCR-2H molar ratio, and the ^1^H NMR spectra of these solutions were recorded. The resulting spectra between 3–10 ppm are shown in Figure 5. The proton signals of the coordinated NiCR-2H were not observable because it is paramagnetic. The relaxations of several proton signals of 5′-dGMP as a function of the concentration of NiCR-2H were observed, and the relaxations were distance dependent. The H-8 at 8.2 ppm had the strongest relaxation response to the concentration of NiCR-2H (Figure 5). The relatively weak relaxations of the H-1′ at 6.3 ppm and the H-5′at 3.9 ppm were also observed (Figure 5). Because the H-8 is the most proximate proton to the coordinated paramagnetic NiCR-2H as compared to the H-1′ and the H-5′ (Scheme 1), its signal has the most influence from NiCR-2H. Interestingly, a previous result from Burrows' group showed that the relaxations of protons were minimal when incubating NiCR with 5′-dGMP [17]. Together with their result, we conclude that NiCR-2H coordinates more strongly with the N-7 of 5′-dGMP than NiCR. The difference in coordination strength of NiCR and NiCR-2H with 5′-dGMP may result from their structural properties. According to the electronic spectra, NiCR-2H has more charge transfer in nature than NiCR [20]. The charge transfer from metal to the isolated imine in NiCR-2H could make its metal center more positive, enhancing the coordination with ligands. It is also well known that the coordination of metal complexes with guanine can promote the oxidation of the complexed guanine. Because NiCR-2H coordinates with guanine more strongly than NiCR, we predict that NiCR-2H should more readily oxidize guanine. The oxidation of guanine by NiCR-2H and NiCR will be discussed in more detail in the next section.

### 3.4. NiCR-2H Oxidizes Guanines More Efficiently than NiCR

**Figure 6:**
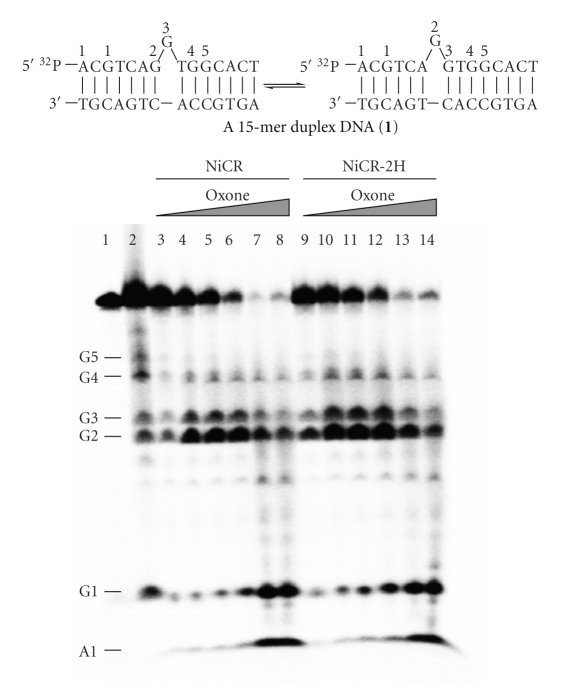
Autoradiogram demonstrating cleavage of **1** by NiCR (30 *μ*M) and NiCR-2H (30 *μ*M). Lane 1: intact DNA; Lane 2: Maxam-Gilbert G-sequencing of **1**; Lane 3–8: DNA incubated with NiCR in the presence of 0.1, 0.2, 0.4, 0.75, 1 mM KHSO_5_, respectively; Lane 9–14: DNA incubated with NiCR-2H in the presence of 0.1, 0.2, 0.4, 0.75, 1 mM KHSO_5_, respectively.

NiCR can selectively oxidize guanines present in distinctive DNA structures such as bulges and loops in the presence of an oxidizing agent [18]. Information on DNA damage by NiCR-2H to our knowledge is very limited. Hence, a side-by-side comparison of DNA damage by these two nickel complexes can be a useful addition to this field and may provide evidence to address our inquiry about the previously observed differences in cytotoxicity. We chose a 15-mer 5′  ^32^P-labeled DNA oligonucleotide duplex containing guanines in a bulge region (**1**, Figure 6) for the DNA cleavage studies because its reactions with NiCR in the presence of KHSO_5 _have already been characterized [36]. In our experiments, no noticeable DNA damage was detected when incubating NiCR or NiCR-2H with **1** in the absence of KHSO_5_ at room temperature for 30 min (See Figure S-4 of the Supplementary Material). KHSO_5_ is proved to be a necessity to produce detectable amounts of DNA damage products under the same conditions. Like NiCR, NiCR-2H in the presence of KHSO_5 _ could not directly produce strand breaks in DNA. However, it undoubtedly damaged DNA because strand breaks (faster moving DNA cleavage products) were detected by gel electrophoresis after treatment of reacted **1** with hot piperidine. The overall cleavage patterns of **1** produced by NiCR-2H are similar to those by NiCR (Figure 6). The Maxam-Gilbert [37] lane (lane 2, Figure 6) shows that these observed migrating bands represent the DNA scission at the guanine residues of **1**. In the presence of KHSO_5_ varied from 100 to 500 *μ*M, the most abundant DNA fragments produced by both NiCR and NiCR-2H were at G2 and G3 in the bulge region (lane 3–6 for NiCR and lane 9–12 for NiCR-2H, Figure 6). Interestingly, a substantially greater amount of cleavage product at G2 was detected compared with that at G3 in both cases (Figure 6). G2 prefers to remain in the bulge in the equilibrium of two bulge conformers (Figure 6) [36]; therefore, it is more prone to oxidation. The amounts of cleavage products of **1** produced by NiCR and NiCR-2H are listed as a bar graph in Figure S-5 of the Supplementary Material. For instance, in the presence of KHSO_5_ (200 *μ*M), the amounts of cleavage products at G2 and G3 by NiCR were (22.3 ± 1.3)% and (11.4 ± 0.9)% and the amounts of cleavage products at G2 and G3 by NiCR-2H were (29.4 ± 0.9)% and (16.5 ± 1.2)%, respectively. NiCR-2H in general provides 5%–9% more of damaged guanine products than NiCR (Figure 6), suggesting that NiCR-2H more readily oxidizes guanine than NiCR. When the concentration of KHSO_5_ was raised above 500 *μ*M, the cleavage products at G1 and A1 became dominant. For instance, in the presence of KHSO_5_ (1 mM), the sums of the cleavage products at G1 and A1 for NiCR and NiCR-2H were (57.4 ± 4.8)% and (68.4 ± 1.6)%, respectively. We believe the changes in damage sites result from the destabilization of **1** by the high concentration of KHSO_5_. This destabilization effect was confirmed by circular dichroism (See Figure S-6 of the Supplementary Material). The destabilization of **1** was only observed when NiCR and KHSO_5_ were both present in the solutions, and KHSO_5 _ alone had no effect on the stability of **1 **(Figure S-7 of the Supplementary Material). When **1** dissociates into random coils, the bulge region no longer exists. In the random coils, G1 and A1 located at the end of the DNA are less well protected than G2 and G3 in the middle of the DNA; therefore, the nickel complexes mainly hit on the less-protected nucleobases. The oxidation of adenine (A1) observed in our experiments has not previously been reported; however, A1 could simply be overoxidized by the large excess of KHSO_5_. Our results for the first time directly compare the efficiency of NiCR and NiCR-2H to oxidize DNA. Both complexes mainly oxidize guanines present in the bulge of **1** in the presence of KHSO_5_, and NiCR-2H more readily oxidizes guanines than NiCR. The oxidation potentials of NiCR and NiCR-2H should not be responsible for their difference in guanine oxidation because both complexes have similar oxidation potential values (1.03 V versus Ag/Ag^+^ for NiCR and 1.05 V versus Ag/Ag^+^ for NiCR-2H, in CH_3_CN) as previously determined in [20]. In fact, the better guanine oxidation by NiCR-2H might be attributable to its stronger coordination with guanines as described in ^1^H NMR. 

Because the cytotoxicity was determined by incubating NiCR or NiCR-2H with cultured cells in the absence of any exogenous oxidant, we then investigated the DNA damage by NiCR or NiCR-2H in the absence of KHSO_5_ at the physiological temperature with a prolonged incubation time. DNA **1** was incubated with either NiCR or NiCR-2H at various concentrations (30, 300, and 600 *μ*M) in the absence of KHSO_5_ at 37°C for 18 h followed by hot piperidine treatment. The DNA cleavage products obtained under this reaction condition was only 1%–5%, which is much less as compared to those obtained in the presence of KHSO_5_. The Maxam-Gilbert method confirmed that both complexes still mainly oxidized guanines in **1** but with no preference to G2 and G3 in the bulge region. (See Figure S-8 of the Supplementary Material). NiCR-2H produced ~2–3.5% more cleavage products (the sum of all product bands) than NiCR. The minimal DNA oxidation by NiCR and NiCR-2H in the absence of KHSO_5_ seems not to be responsible for the observed differences in cytotoxicity to MCF-7 cells. However, this conclusion is drawn without taking endogenous oxidants into consideration. Endogenous oxidants such as reactive oxygen species (ROSs) are known to promote the DNA damage induced by metal complexes [38, 39]. NiCR and NiCR-2H in cultured cells in principle could efficiently oxidize guanines in the presence of endogenous oxidant(s), leading to the differences in cytotoxicity.

### 3.5. NiCR Is Oxidized into NiCR-2H by KHSO_5_ at Low Ligand-Oxidant Ratios

**Figure 7:**
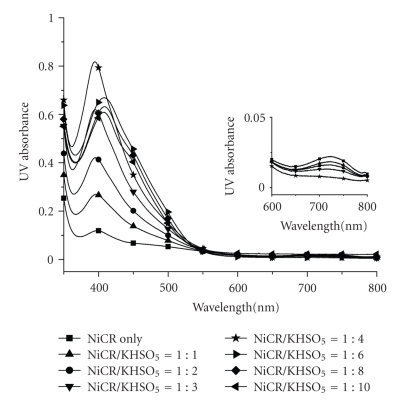
Absorption spectra of NiCR (1 mM) at different NiCR/KHSO_5_ ratios. Experimental conditions: water at 25°C. Insert: The zoom-in region of the absorption spectra between 600–800 nm.

Previous studies on oxidation of DNA by NiCR always adopted high-ligand oxidant ratios [18]. A Ni (III) complex was also proposed as an important intermediate for oxidation of guanine [15]. Because the cytotoxicity of NiCR-2H was observed without any exogenous oxidant, it is necessary to study oxidation of the nickel complexes at low-oxidative stress conditions. Oxidation of NiCR without an exogenous oxidant is very slow and therefore is not suitable for study. The oxidation of NiCR by KHSO_5_ with different molar ratios to NiCR was first investigated using UV absorption spectroscopy. The spectrum of NiCR showed a maximum absorption at 399 nm and a weak absorption at 720 nm (Figure 7). Because of its low extinction coefficient, 1 mM NiCR was used for this study. The maximum absorption (*λ*_max _) increased dramatically as a function of the concentration of KHSO_5 _ ranging from 0 to 4 mM (KHSO_5_/NiCR ≤ 4). A blue shift of *λ*_max_ from 399 nm to 394 nm was observed (Figure 7). The weak absorption at 720 nm in the spectrum of NiCR also decreased accordingly (Figure 7, insert). The changes in the UV spectra indicate the oxidation of NiCR. Interestingly, the UV spectrum of this newly formed oxidation product is very similar to that of NiCR-2H, which also has a *λ*_max_ at 394 nm and no absorption at 720 nm as well. When the concentration of KHSO_5_ was over 4 mM (KHSO_5_/NiCR > 4), the absorption at 394 nm decreased accompanying a red shift of 14 nm to *λ*_max_ at 408 nm, suggesting that a secondary oxidation occurred (Figure 7).

**Figure 8:**
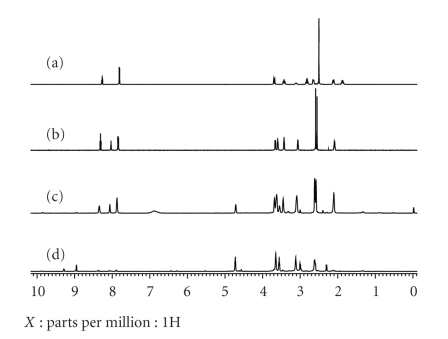
^1^H NMR spectra of NiCR (a), NiCR-2H (b), oxidation product of NiCR with 3 equivalents of KHSO_5_ (c), and oxidation product of NiCR with 10 equivalents of KHSO_5_ (d).

Additional support for the oxidation of NiCR into NiCR-2H at low KHSO_5_/NiCR ratios (≤4) was gleaned from ^1^H NMR. The diamagnetic NMR spectra of NiCR and NiCR-2H were obtained using CF_3_COOD as a solvent, suggesting that CF_3_COOD is a weak ligand that cannot form a paramagnetic complex with NiCR or NiCR-2H. The reactions of NiCR with 3 or 10 equivalents of KHSO_5_ were carried out, and the resulting products were measured by ^1^H NMR. The spectra of NiCR, NiCR-2H, and the oxidation products are shown in Figure 8. The spectrum (Figure 8(c)) of the product obtained from the reaction of NiCR with 3 equivalents of KHSO_5_ is very similar to that of NiCR-2H (Figure 8(b)). The signal at 8.2 ppm clearly indicates the formation of the imine group. In addition, the methyl protons of the product appear as two 1 : 1 singlet peaks at 2.60 and 2.62 ppm, suggesting an asymmetrical structure. In contrast, the methyl protons of NiCR appear as a singlet at 2.5 ppm (Figure 8(a)) because of its symmetrical structure. Oxidation of NiCR with 10 equivalents of KHSO_5_ gave a ^1^H NMR spectrum containing no signals between 7.5 and 8.5 ppm, which is completely different from that of NiCR or NiCR-2H (Figure 8(d)). The results presented here are direct evidence for oxidation of NiCR into NiCR-2H by KHSO_5_ under physiological conditions, which have not been previously reported. Because such oxidation occurs at low oxidant/ligand molar ratios, we believe that it is possible to oxidize NiCR into NiCR-2H *in vivo* in the absence of an exogenous oxidant. NiCR-2H is relatively stable under physiological conditions and may survive a long period of time in cultured cells. The role of NiCR-2H in oxidation of guanine by NiCR may be underestimated in previous studies, and NiCR-2H could be an important precursor for the proposed Ni (III) intermediate.

## 4. Conclusions

Amongst three cancer cell lines, NiCR-2H is only cytotoxic to MCF-7 cells (IC50: 20 *μ*M) and both NiCR and NiCR-2H have neglectable effect on HeLa and A549. In order to understand the differences in cytotoxicity, we in this paper have investigated the interactions of NiCR and NiCR-2H with DNA. We conclude that the differences in cytotoxicity should not result from the differences in the binding of NiCR and NiCR-2H with DNA because both complexes bind weakly in the grooves of DNA with no quantitative differences. Both NiCR and NiCR-2H damage DNA with a similar sequence preference, and NiCR-2H more readily oxidizes guanine than NiCR in the presence of KHSO_5_ probably due to its stronger coordination with guanine. The differences in oxidation of guanine between NiCR and NiCR-2H could be a key to the differences in cytotoxicity. However, this is not conclusive because the role of exogenous oxidants is unknown. We have also obtained the direct evidences for oxidation of NiCR into NiCR-2H at low molar ratios of KHSO_5_/NiCR, suggesting NiCR-2H could act as an important precursor for the previously proposed Ni (III) intermediate. The investigation of molecular interactions of NiCR and NiCR-2H with DNA is the first step toward understanding the differences in cytotoxicity. The ultimate explanation on this matter must be more complicated and requires understanding of the biological responses of NiCR and NiCR-2H *in vivo* such as cellular uptake and cellular metabolism.

## Supplementary Material

Details of experimental procedures and spectra for UV denaturation of DNA oligonucleotide duplexes with NiCR and NiCR-2H, circular dichroism titration of 1 with oxone, and DNA cleavage studies under various conditions, and dye exclusion studies. This material is available online on doi: 10.1155/2010/619436.
