# Supplementary material for: Investigation on the Interactions of NiCR and NiCR-2H with DNA
Source: Bioinorg Chem Appl. 2010 Jun 30;2010:619436. doi: 10.1155/2010/619436 (PMC2910472; doi:10.1155/2010/619436)
Supplement: Supplementary file 1 — Details of experimental procedures and spectra for UV denaturation of DNA oligonucleotide duplexes with NiCR and NiCR-2H, circular dichroism titration of 1 with oxone, and DNA cleavage studies under various conditions, and dye exclusion studies. This material is available online on doi: 10.1155/2010/619436. [file 619436.f1.pdf]

Supporting Information: Investigation on the interactions of NiCR and NiCR-2H with DNA

Priyanka Chitranshi, Chang-Nan Chen, Patrick R. Jones, Jesika S. Faridi, and Liang Xue
Department of Chemistry, University of the Pacific, Stockton, California 95211

Contents:

1. Experimental Procedures.
2. **Supporting Information Figure S-1.** UV melting curves of a 22 mer AT-tract DNA oligonucleotide duplex with NiCR at various concentrations.
3. **Supporting Information Figure S-2.** UV melting curves of a 22 mer AT-tract DNA oligonucleotide duplex with NiCR-2H at various concentrations.
4. **Supporting Information Figure S-3.** UV melting curves of a 16 mer GC-rich DNA oligonucleotide duplex with NiCR-2H at various concentrations.
5. **Supporting Information Figure S-4.** Yield of DNA cleavage products obtained in the reaction of **1** with NiCR (A) and NiCR-2H (B) in the presence of $KHSO_5$.
6. **Supporting Information Figure S-5.** Autoradiogram demonstrating reaction of **1** under various control conditions.
7. **Supporting Information Figure S-6.** Reaction of **1** with NiCR and NiCR-2H at 37 °C overnight in the absence of oxone.
8. **Supporting Information Figure S-7.** Circular dichroism spectra of **1** in the presence of NiCR (30 μM) with $KHSO_5$ at various concentrations.
9. **Supporting Information Figure S-8.** Circular dichroism spectra of **1** in the presence of $KHSO_5$ at various concentrations.

Experimental Procedures:

UV denaturation of a 22 mer AT oligonucleotide duplex with NiCR and NiCR-2H. The 22 mer dA (100 μM) and dT (100 μM) was dissolved in a mixture (100 μL) of phosphate buffer (10 mM, pH 7.0) and NaCl (100 mM). The mixture was heated at 90 °C for 5 min, slowly cooling down to 25 °C, and incubated at 4 °C overnight to form the 22 mer AT duplex DNA. The UV denaturation samples (1 mL) were prepared by mixing the duplex DNA (1 μM), phosphate buffer (10 mM, pH 7.0), NaCl (100 mM) and NiCR or NiCR-2H at various concentrations (0, 0.5, 1, 5, 10, and 25 μM). The UV melting spectra of these samples in 1 cm path length Quartz cuvettes was recorded at 260 nm as a function of temperature (10-95 °C, heating rate: 0.5 °C/min).

The UV denaturation of a 16 mer GC-rich oligonucleotide duplex with NiCR-2H was performed similarly. The DNA sequence used was 5′-GAAGACCTGGGCGTCC along with its complementary strand to form a duplex.

Circular dichroism titration. To a mixture (1.8 mL) of **1** (5 μM) and NiCR (30 μM) in phosphate buffer (10 mM, pH 7.0) and NaCl (100 mM) at 20 °C, was added small aliquots of oxone stock solution to reach the desired oxone concentrations (0, 0.1, 0.2, 0.3, 0.5, 0.75, and 1 mM) of $KHSO_5$, respectively. After each addition, the mixture was incubated for 20 min at 20 °C prior to the measurements. All of the circular dichroism (CD) spectra were collected from 200 to 350 nm. The CD spectra of **1**, a 15 mer single-stranded DNA oligonucleotide [**2**, 5’-d(ACGTCAGGTGGCACT)], and **1** in the presence of $KHSO_5$ (0-1 mM) were recorded as references.

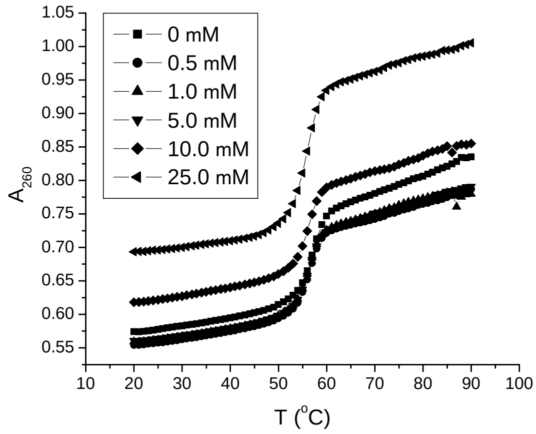

Concentration (μM) of NiCR	T_m (°C)
0	57.02
0.5	57.12
1.0	56.22
5.0	56.27
10.0	56.32
25.0	56.32

Figure S-1. UV melting curves of a 22 mer AT-tract DNA oligonucleotide duplex in the presence of NiCR at various concentrations. [DNA] = 1 μM; [NiCR] = 0, 0.5, 1, 5, 10, and 25 μM. Experimental conditions: phosphate buffer (10 mM) at pH 7.0, and NaCl (100 mM). The Y axis is offset for a clear presentation.

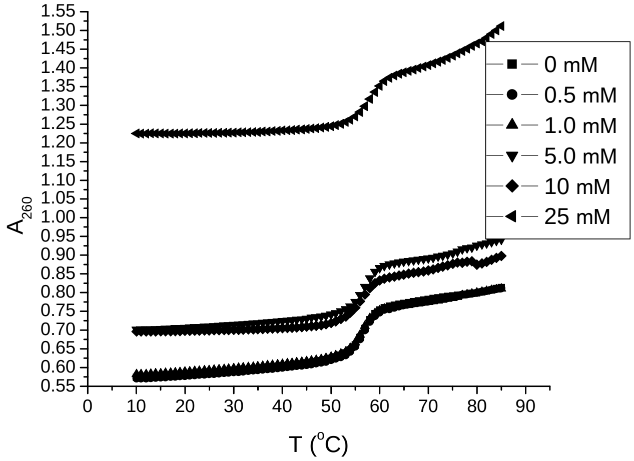

Concentration (μM) of NiCR-2H	T_m (°C)
0	57.02
0.5	57.17
1.0	56.27
5.0	57.32
10.0	56.36
25.0	58.37

Figure S-2. UV melting curves of a 22 mer AT-tract DNA oligonucleotide duplex in the presence of NiCR-2H at various concentrations. [DNA] = 1 μM; [NiCR-2H] = 0, 0.5, 1, 5, 10, and 25 μM. Experimental conditions: phosphate buffer (10 mM) at pH 7.0, and NaCl (100 mM). The Y axis is offset for a clear presentation.

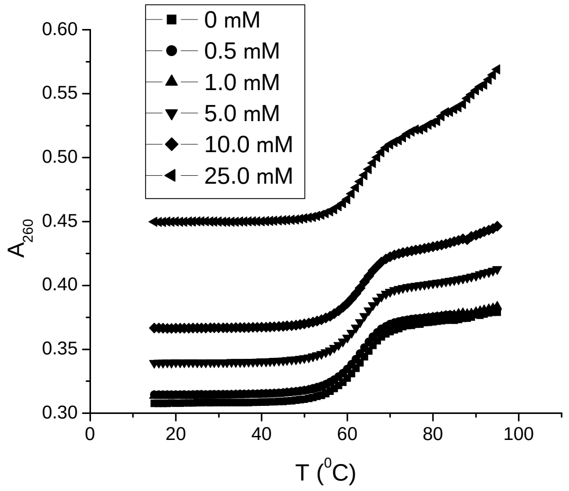

Concentration (μM) of NiCR-2H	T_m (°C)
0	62.02
0.5	62.07
1.0	62.07
5.0	62.12
10.0	62.17
25.0	62.22

Figure S-3. UV melting curves of a 16 mer GC-rich DNA oligonucleotide duplex in the presence of NiCR-2H at various concentrations. [DNA] = 1 μM; [NiCR-2H] = 0, 0.5, 1, 5, 10, and 25 μM. Experimental conditions: phosphate buffer (10 mM) at pH 7.0, and NaCl (100 mM). The Y axis is offset for a clear presentation.

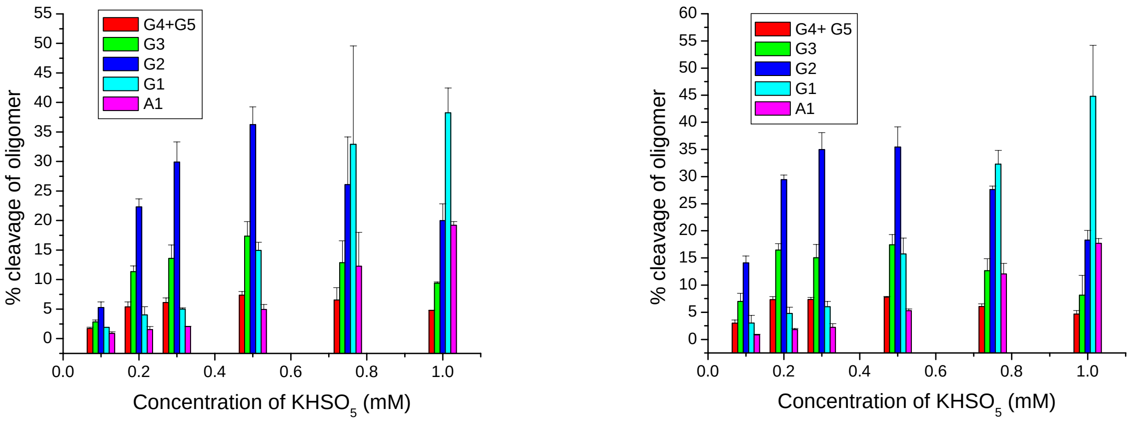

A B

Figure S-4. Yield of DNA cleavage products obtained in the reaction of **1** with NiCR (A) and NiCR-2H (B) in the presence of $KHSO_5$. This graph refers to Figure 8 of the main text.

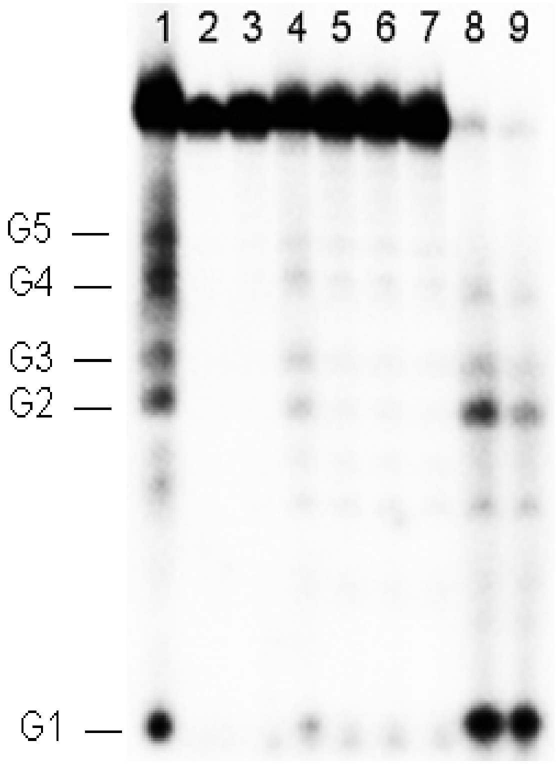

Figure S-5. Autoradiogram demonstrating reaction of **1** under various control conditions. Lane 1: Maxam Gilbert G sequencing of **1**. Lane 2: intact **1**. Lane 3: **1** incubated with $KHSO_5$ (1 mM) and no piperidine treatment. Lane 4: **1** incubated with $KHSO_5$ (1 mM) followed by hot piperidine treatment. Lane 5: **1** incubated with hot piperidine treatment only. Lane 6: **1** incubated with NiCR (30 μM) in the absence of oxone followed by hot piperidine treatment. Lane 7: **1** incubated with NiCR-2H (30 μM) in the absence of oxone followed by hot piperidine treatment. Lane 8: **1** incubated with NiCR (30 μM) and $KHSO_5$ (1 mM) followed by hot piperidine treatment. Lane 9: **1** incubated with NiCR-2H (30 μM) and $KHSO_5$ (1 mM) followed by hot piperidine treatment. The incubation time for all the reactions was 30 min.

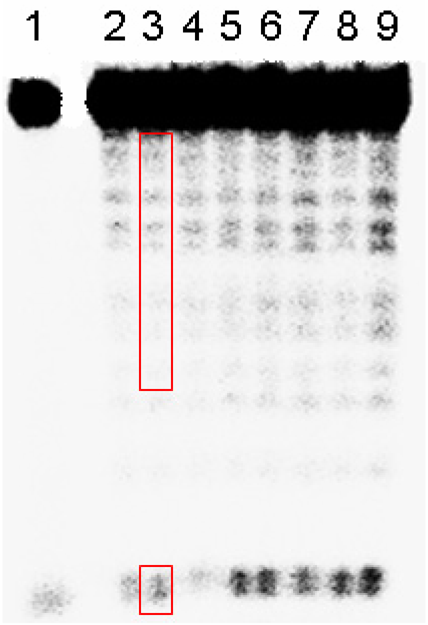

A

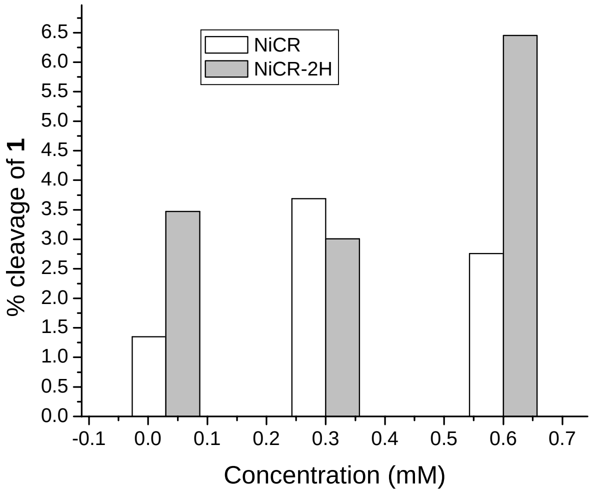

B

Figure S-6. Reaction of **1** with NiCR or NiCR-2H in the absence of oxone at 37 °C overnight. A: Autoradiogram demonstrating reaction of **1** with NiCR or NiCR-2H incubated at 37 °C overnight in the absence of oxone. Lane 1: intact DNA. Lane 2: **1** incubated with NaCl (100 mM) and phosphate buffer (10 mM, pH 7.0) for 30 min. Lane 3: **1** incubated with NaCl (100 mM) and phosphate buffer (10 mM, pH 7.0) overnight. Lane 4-6: **1** incubated with NiCR at 0.03, 0.3, and 0.6 mM overnight. Lane 7-9: **1** incubated with NiCR-2H at 0.03, 0.3, and 0.6 mM NiCR-2H overnight. B: Graphical representation of the sum of DNA cleavage products (the rectangular region in A) derived from A.

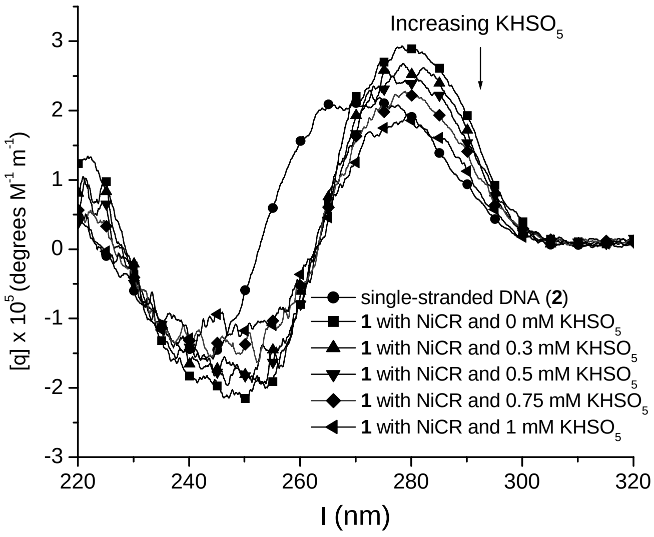

Figure S-7. Circular dichroism spectra of **1** in the presence of NiCR (30 μM) and $KHSO_5$ at various concentrations. [$KHSO_5$] = 0, 0.3, 0.5, 0.75, and 1 mM. [DNA] = 5 μM. Experimental conditions: phosphate buffer (10 mM) at pH 7.0, and NaCl (100 mM). The spectrum of **2** is shown as reference.

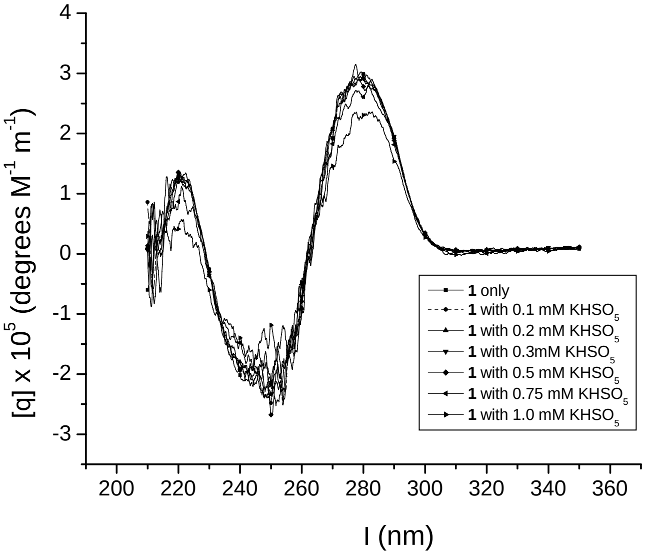

Figure S-8. Circular dichroism spectra of **1** in the presence of $KHSO_5$ at various concentrations. [$KHSO_5$] = 0, 0.3, 0.5, 0.75, and 1 mM. [DNA] = 5 μM. Experimental conditions: phosphate buffer (10 mM) at pH 7.0, and NaCl (100 mM).
